# Supplementary material for: Mutations in PCYT2 disrupt etherlipid biosynthesis and cause a complex hereditary spastic paraplegia
Source: Brain. 2019 Oct 22;142(11):3382–97. doi: 10.1093/brain/awz291 (PMC6821184; doi:10.1093/brain/awz291)
Supplement: awz291_Supplementary_Data [file awz291_supplementary_data.zip › awz291-Suppl_data/Supplementary_Data4.zip!/Supplementary information 4 - plasma lipidomics.zip!/Plasma lipidomics/Control vs Patient3a/Statistical plots and data/Heatmap sorted on FDR.pdf]

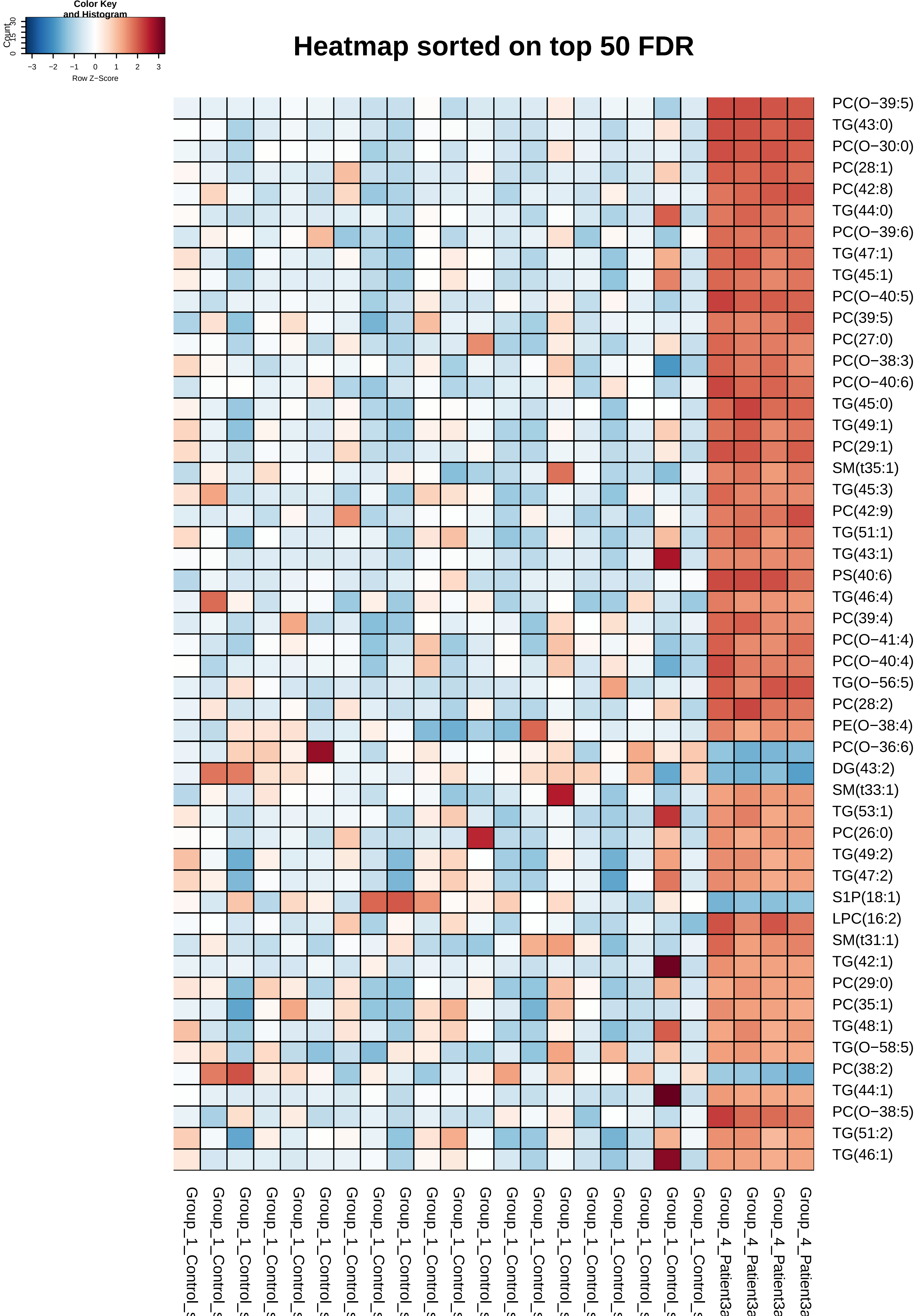
Color Key
and Histogram
Count
0
15
30
−3
−2
−1
0
1
2
3
Row Z−Score
Heatmap sorted on top 50 FDR
PC(O−39:5)
TG(43:0)
PC(O−30:0)
PC(28:1)
PC(42:8)
TG(44:0)
PC(O−39:6)
TG(47:1)
TG(45:1)
PC(O−40:5)
PC(39:5)
PC(27:0)
PC(O−38:3)
PC(O−40:6)
TG(45:0)
TG(49:1)
PC(29:1)
SM(t35:1)
TG(45:3)
PC(42:9)
TG(51:1)
TG(43:1)
PS(40:6)
TG(46:4)
PC(39:4)
PC(O−41:4)
PC(O−40:4)
TG(O−56:5)
PC(28:2)
PE(O−38:4)
PC(O−36:6)
DG(43:2)
SM(t33:1)
TG(53:1)
PC(26:0)
TG(49:2)
TG(47:2)
S1P(18:1)
LPC(16:2)
SM(t31:1)
TG(42:1)
PC(29:0)
PC(35:1)
TG(48:1)
TG(O−58:5)
PC(38:2)
TG(44:1)
PC(O−38:5)
TG(51:2)
TG(46:1)
Group_1_Control_
Group_1_Control_
Group_1_Control_
Group_1_Control_
Group_1_Control_
Group_1_Control_
Group_1_Control_
Group_1_Control_
Group_1_Control_
Group_1_Control_
Group_1_Control_
Group_1_Control_
Group_1_Control_
Group_1_Control_
Group_1_Control_
Group_1_Control_
Group_1_Control_
Group_1_Control_
Group_1_Control_
Group_1_Control_
Group_4_Patient3a
Group_4_Patient3a
Group_4_Patient3a
Group_4_Patient3a
